# Provocation of migraine with aura using natural trigger factors

**DOI:** 10.1186/1129-2377-14-S1-P110

**Published:** 2013-02-21

**Authors:** A Hougaard, FM Amin, AW Hauge, M Ashina, J Olesen

**Affiliations:** 1 Danish Headache Center, Department of Neurology, University of Copenhagen, Faculty of Health Sciences, Denmark

## Objective

It is well known that migraine attacks can be precipitated by various stimuli. More than 50% of migraine with aura (MA) patients know of at least one stimulus that always or often triggers their MA attacks. The objective of this study was to expose MA patients to their self-reported trigger factors in order to assess the causal relation between trigger factors and attacks.

## Methods

We recruited 27 MA patients who reported that bright or flickering light and/or strenuous exercise would trigger their migraine attacks. The patients were experimentally provoked by either different types of photo stimulation, strenuous exercise or a combination of these two factors. During and following provocation the patients would report any aura symptoms or other migraine related symptoms.

## Results

Of 27 provoked MA patients 3 (11%) reported attacks of MA following provocation. An additional 3 patients reported MO attacks. Following exercise, 4 out of 12 patients reported migraine, while no patients developed attacks following photo stimulation.

## Conclusions

Experimental provocation using self-reported natural trigger factors causes MA only in a small subgroup of MA patients. Prospective confirmation is important for future studies of migraine trigger factors and in the clinical management of migraine patients.
